# Supplementary material for: An open-source framework for large-scale, flexible evaluation of biomedical text mining systems
Source: J Biomed Discov Collab. 2008 Jan 29;3:1. doi: 10.1186/1747-5333-3-1 (PMC2276192; doi:10.1186/1747-5333-3-1)
Supplement: Additional file 1 — Evaluation platform source code. The additional file contains the software evaluation framework discussed in this paper. The most current release can be downloaded from SourceForge.net [1]. See the accompanying README file for instructions on installation and use. Also included in the distribution is a collection of UIMA wrappers for some commonly used BioNLP tools and annotated corpora. [file 1747-5333-3-1-S1.zip › bionlp_uima_repository-1.0.1/docs/api/edu/uchsc/ccp/util/nlp/annotation/comparison/AnnotationComparator.html]

AnnotationComparator (Center for Computational Pharmacology Util/UIMA API)


|  |  |  |
| --- | --- | --- |
| **Overview** \| **Package** \| **Class** \| **Tree** \| **Deprecated** \| **Index** \| **Help** | |  |
| PREV CLASS   **NEXT CLASS** | **FRAMES**    **NO FRAMES**     **All Classes** |  |
| SUMMARY: NESTED \| FIELD \| CONSTR \| METHOD | DETAIL: FIELD \| CONSTR \| METHOD |  |


---


## edu.uchsc.ccp.util.nlp.annotation.comparison Class AnnotationComparator

```
java.lang.Object
  edu.uchsc.ccp.util.nlp.annotation.comparison.AnnotationComparator
```

**All Implemented Interfaces:**: Comparator<TextAnnotation>

**Direct Known Subclasses:**: EntityPartsAnnotationComparator

---

``` public class AnnotationComparator extends Object implements Comparator<TextAnnotation> ```

---

**Constructor Summary**

`AnnotationComparator()`


| **Method Summary** | |
| --- | --- |
| `PRFResult` | `compare(List<TextAnnotation> goldTaList, List<TextAnnotation> taList)`             Default comparison of two lists of TextAnnotations utilizing the StrictSpanComparator and the IdenticalMentionComparator family |
| `PRFResult` | `compare(List<TextAnnotation> goldTaList, List<TextAnnotation> taList, SpanComparator spanComparator, ClassMentionComparator classMentionComparator)`             Compares two lists of TextAnnotations using the supplied SpanComparator and ClassMentionComparator |
| `PRFResult` | `compare(List<TextAnnotation> goldTaList, List<TextAnnotation> testTaList, SpanComparator spanComparator, ClassMentionComparator classMentionComparator, int maximumComparisonDepth)`             Compares two lists of TextAnnotations using the supplied SpanComparator and ClassMentionComparator, but only compares to the supplied comparison depth. |
| `int` | `compare(TextAnnotation ta1, TextAnnotation ta2)`             Default TextAnnotation comparison utilizes the StrictSpanComparator and the IdenticalMentionComparator family |
| `int` | `compare(TextAnnotation ta1, TextAnnotation ta2, SpanComparator spanComparator, ClassMentionComparator classMentionComparator)`             Compares two TextAnnotations using the supplied SpanComparator and ClassMentionComparator |
| `int` | `compare(TextAnnotation ta1, TextAnnotation ta2, SpanComparator spanComparator, ClassMentionComparator classMentionComparator, int maximumComparisonDepth)`             Compares two TextAnnotations using the supplied SpanComparator and ClassMentionComparator, but only compares to the supplied comparison depth. |
| `int` | `metaDataCompare(TextAnnotation ta1, TextAnnotation ta2)`             Compare the meta data of two annotations (document ID, document collection ID, and span(s)). |

**Methods inherited from class java.lang.Object**

`clone, equals, finalize, getClass, hashCode, notify, notifyAll, toString, wait, wait, wait`

**Methods inherited from interface java.util.Comparator**

`equals`

**Constructor Detail**

### AnnotationComparator

```
public AnnotationComparator()
```


**Method Detail**

### compare

```
public int compare(TextAnnotation ta1,
                   TextAnnotation ta2)
```

:   Default TextAnnotation comparison utilizes the StrictSpanComparator and the IdenticalMentionComparator family

    :   **Specified by:**: `compare` in interface `Comparator<TextAnnotation>`

---


### compare

```
public int compare(TextAnnotation ta1,
                   TextAnnotation ta2,
                   SpanComparator spanComparator,
                   ClassMentionComparator classMentionComparator)
```

:   Compares two TextAnnotations using the supplied SpanComparator and ClassMentionComparator

    :   **Parameters:**: `ta1` -: `ta2` -: `spanComparator` -: `classMentionComparator` - **Returns:**

---


### compare

```
public PRFResult compare(List<TextAnnotation> goldTaList,
                         List<TextAnnotation> taList)
```

:   Default comparison of two lists of TextAnnotations utilizing the StrictSpanComparator and the IdenticalMentionComparator family

    :   **Parameters:**: `taList1` -: `taList2` - **Returns:**

---


### compare

```
public PRFResult compare(List<TextAnnotation> goldTaList,
                         List<TextAnnotation> taList,
                         SpanComparator spanComparator,
                         ClassMentionComparator classMentionComparator)
```

:   Compares two lists of TextAnnotations using the supplied SpanComparator and ClassMentionComparator

    :   **Parameters:**: `taList1` -: `taList2` -: `spanComparator` -: `classMentionComparator` - **Returns:**

---


### compare

```
public int compare(TextAnnotation ta1,
                   TextAnnotation ta2,
                   SpanComparator spanComparator,
                   ClassMentionComparator classMentionComparator,
                   int maximumComparisonDepth)
```

:   Compares two TextAnnotations using the supplied SpanComparator and ClassMentionComparator, but only compares to the supplied comparison depth.

    :   **Parameters:**: `ta1` -: `ta2` -: `spanComparator` -: `mentionComparator` - **Returns:**

---


### compare

```
public PRFResult compare(List<TextAnnotation> goldTaList,
                         List<TextAnnotation> testTaList,
                         SpanComparator spanComparator,
                         ClassMentionComparator classMentionComparator,
                         int maximumComparisonDepth)
```

:   Compares two lists of TextAnnotations using the supplied SpanComparator and ClassMentionComparator, but only compares to the supplied
    comparison depth.

    :   **Parameters:**: `taList1` -: `taList2` -: `spanComparator` -: `classMentionComparator` -: `maximumComparisonDepth` - **Returns:**

---


### metaDataCompare

```
public int metaDataCompare(TextAnnotation ta1,
                           TextAnnotation ta2)
```

:   Compare the meta data of two annotations (document ID, document collection ID, and span(s)). A warning is issued if it is determined that two
    annotations do not have identical meta data

    :   **Parameters:**: `ta` - **Returns:**


---


|  |  |  |
| --- | --- | --- |
| **Overview** \| **Package** \| **Class** \| **Tree** \| **Deprecated** \| **Index** \| **Help** | |  |
| PREV CLASS   **NEXT CLASS** | **FRAMES**    **NO FRAMES**     **All Classes** |  |
| SUMMARY: NESTED \| FIELD \| CONSTR \| METHOD | DETAIL: FIELD \| CONSTR \| METHOD |  |


---
